# The Potential Role of Quantum Computing in Biomedicine and Healthcare: The Next Frontier Beyond Artificial Intelligence

**DOI:** 10.7759/cureus.82759

**Published:** 2025-04-22

**Authors:** Tarun Kumar Suvvari, Venkata Sai Bhargav Pradeep Konakanchi, Ramya Sree Muppavarapu, Nithya Arigapudi

**Affiliations:** 1 General Medicine, Rangaraya Medical College, Kakinada, IND; 2 Research, Squad Medicine and Research (SMR), Andhra Pradesh, IND; 3 Emergency Medicine, Midland Metropolitan University Hospital, Birmingham, GBR; 4 Medicine, Andhra Medical College, Visakhapatnam, IND; 5 Internal Medicine, Konaseema Institute of Medical Sciences, Vijayawada, IND; 6 Medicine, Dr. Pinnamaneni Siddhartha Institute of Medical Sciences & Research Foundation, Vijayawada, IND

**Keywords:** artificial intelligence, biomedicine, digital health communications, medical and healthcare, quantum computing

## Abstract

Quantum computing is poised to revolutionize biomedicine and healthcare, offering computational advantages that surpass classical and artificial intelligence-based approaches. Its ability to process complex biological data, simulate molecular interactions, and optimize drug discovery presents unprecedented opportunities for personalized medicine and disease modeling. Quantum algorithms can enhance genomic analysis, accelerating diagnostics and therapeutic interventions. Quantum-enhanced machine learning may refine predictive models for patient outcomes and epidemiological trends. Despite its transformative potential, challenges such as hardware limitations, error rates, and algorithm development must be addressed for practical implementation. This editorial explores the emerging role of quantum computing in biomedical research and healthcare, highlighting its capabilities, current advancements, and future implications.

## Editorial

While artificial intelligence (AI) is currently transforming biomedicine and healthcare, quantum computing (QC) represents an even more revolutionary paradigm shift. QC offers unprecedented capabilities for tackling complex problems that are currently beyond the reach of classical computers, potentially transforming medical research and clinical practice [1]. By applying quantum principles, researchers are developing more precise diagnostic tools, enhancing personalized treatment plans, and gaining deeper insights into disease mechanisms, marking a new era in medicine [2].

The promising role of quantum computing

Traditional computers use bits to represent information as 0 or 1, while quantum computers use qubits. Qubits can exist in a state of superposition, representing 0, 1, or both simultaneously, and entanglement, where multiple qubits are linked [3]. This enables quantum computers to perform calculations in parallel, processing vast amounts of data exponentially faster than classical computers [3]. This computational power is particularly suited for handling the massive datasets and complex simulations inherent in biomedicine and healthcare [2,3].

Transforming healthcare applications

**Figure 1:**
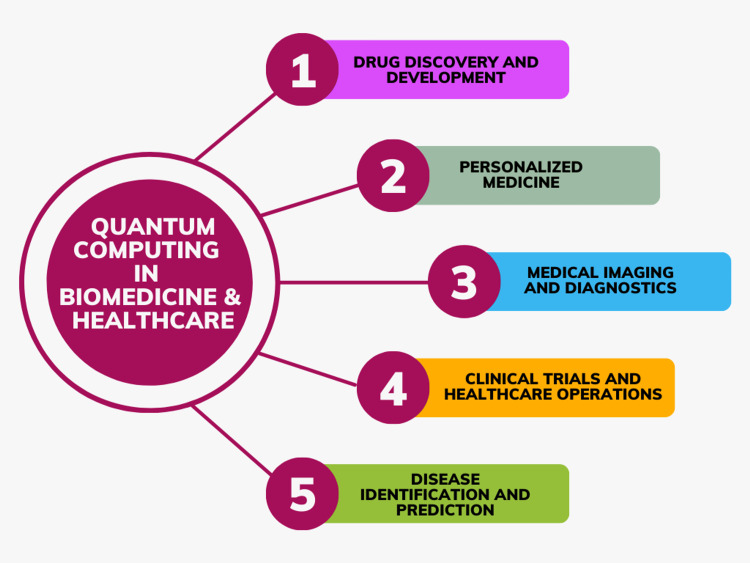
Applications of quantum computing in biomedicine & healthcare Image Credits: Dr.Tarun Kumar Suvvari

QC's ability to process and analyze vast datasets, model complex biological systems, and optimize decision-making processes positions it as a transformative tool in medical research and clinical applications (Figure 1).

Drug Discovery and Development

Quantum computing can significantly enhance early-stage drug discovery by simulating molecular interactions at the quantum level, something that classical computers struggle to do accurately. Traditional methods often rely on approximations that fail to capture the full complexity of atomic and electronic behavior [4]. Quantum algorithms can compute the ground-state energy of molecules, helping researchers understand binding affinities and chemical reactivity with much higher precision. This capability is crucial for predicting how potential drug compounds will interact with target proteins, enabling more efficient structure-based drug design [1,4]. In addition to simulation, quantum computing can process vast chemical libraries in parallel, dramatically accelerating hit identification and lead optimization. Unlike classical high-throughput screening, which is resource-intensive, quantum algorithms can evaluate numerous molecular configurations simultaneously, identifying promising candidates faster and with fewer false leads. This reduces both the time and cost required to bring a drug to market while also improving the likelihood of clinical success [4]. As the technology matures, hybrid quantum-classical models are expected to drive breakthroughs in molecular docking, ADME (absorption, distribution, metabolism, and excretion) prediction, and toxicity profiling, reshaping pharmaceutical Research & Development into a data-driven, predictive science [2,4].

Protein Folding

Understanding protein folding is essential for studying diseases like Alzheimer’s and Parkinson’s, which are related to protein misfolding [5]. Quantum computing can improve the efficiency of predicting protein folding patterns [5]. Quantum computing introduces a new paradigm by enabling the simulation of molecular systems at a quantum-mechanical level, where the interactions between atoms and electrons can be modeled more precisely. Specifically, the quantum approximate optimization algorithm (QAOA) and variational quantum eigensolver (VQE) are two promising quantum algorithms capable of addressing the complex energy landscape of protein folding. These algorithms frame the folding problem as an optimization task, finding the minimum energy conformation among an exponential number of configurations and making them well-suited for implementation on quantum processors [2,5]. Moreover, quantum computing could eventually enable real-time simulation of protein dynamics, offering insights into folding pathways, intermediate states, and misfolding triggers that are otherwise inaccessible. This capability would not only accelerate drug discovery by improving target validation but could also enable the structure-based design of therapeutic molecules tailored to stabilize or refold pathogenic proteins [2,5].

Personalized Medicine

Quantum algorithms can integrate diverse data sources, such as a patient’s genome, medical history, and lifestyle factors, to provide treatment recommendations tailored to each patient [6]. QC can also improve the accuracy of predicting patient responses to drugs based on genetic profiles, potentially revolutionizing how treatments are personalized [2,6]. Quantum-enhanced machine learning can process high-dimensional, multi-omics datasets, such as genomic, transcriptomic, proteomic, and metabolomic profiles, faster and more accurately than classical systems. By capturing the full spectrum of biological complexity, quantum computing could help design personalized therapeutic strategies in oncology, neurology, and rare genetic disorders, shifting healthcare from a reactive to a truly predictive and preventive model.

Medical Imaging and Diagnostics

Quantum systems can enhance the resolution and accuracy of medical imaging techniques like MRIs, CT scans, and ultrasounds [1]. Quantum machine learning can help improve the accuracy or reduce the number of features needed for predictions, for example, it can help in faster image reconstruction, superior data compression, analyzing MRI images, and improved feature extraction [1,2]. From a technical standpoint, quantum algorithms, such as the quantum Fourier transform and quantum-enhanced principal component analysis (qPCA), can be used for advanced image denoising and dimensionality reduction. These enable faster and more precise analysis of high-resolution images with fewer computational resources compared to classical counterparts. For instance, in MRI imaging, QML models trained on quantum hardware can detect anomalies in brain scans with fewer features by exploiting quantum entanglement and superposition for superior pattern recognition [6,7].

Clinical Trials and Healthcare Operations

Quantum computing can transform the design and execution of clinical trials by optimizing complex variables such as patient selection, treatment sequencing, and adaptive randomization [2,7]. Quantum algorithms like the QAOA and quantum-enhanced machine learning allow more precise matching of patients to trial arms based on genomic or phenotypic data. This results in safer, more effective treatments and faster trial completion, directly improving patient outcomes and advancing personalized medicine strategies [7,8]. In broader healthcare operations, quantum algorithms enable intelligent resource allocation and scheduling, helping hospitals optimize bed usage, staff deployment, and equipment availability in real time. By reducing treatment delays, minimizing scheduling conflicts, and streamlining workflows, quantum computing supports more responsive and cost-efficient healthcare delivery. These improvements not only enhance operational performance but also ensure timely, high-quality care for patients enrolled in clinical trials and beyond [7,8].

Disease Identification and Prediction

Quantum-powered AI algorithms can quickly and accurately diagnose diseases by analyzing images and complex data points [2,9]. QC can analyze data from wearable devices, personal health records, pictures, and geographic, environmental, and climate trends, to anticipate sickness before symptoms appear and suggest treatment options and customized healthcare plans [9].

Challenges and future directions

While quantum computing holds immense promise, current quantum hardware remains in its nascent stage. These systems require ultra-low temperatures, isolation from environmental noise, and specialized infrastructure, making them fragile and difficult to scale [3,10,11]. A major technical challenge is quantum decoherence, the process by which quantum states lose their coherence due to interaction with their environment [11]. This leads to a rapid loss of quantum information, severely limiting the time available for computation. Decoherence is often triggered by thermal fluctuations, electromagnetic interference, or imperfect isolation, and can disrupt quantum entanglement - a cornerstone for quantum advantage [1,2,10,11].

To overcome this, researchers are developing quantum error correction (QEC) techniques such as surface codes and topological qubits, which redundantly encode information to detect and correct errors without measuring the quantum state directly. However, these techniques require a large number of physical qubits to support a single logical qubit, increasing hardware complexity [1,2,10,11]. Additionally, the ability of QC to process vast healthcare datasets raises privacy and security concerns. Regulatory compliance is complex, requiring adherence to medical standards and lengthy approval processes [3,10]. Ethical challenges include ensuring equitable access, preventing biases, and maintaining patient autonomy. High costs further hinder widespread adoption, and seamless integration with classical systems is essential for effective implementation in healthcare [3,9-11].

Conclusion

To conclude, although artificial intelligence has already transformed healthcare, quantum computing emerges as the next frontier with the potential to surpass current computational limits in biomedicine and healthcare. From revolutionizing drug discovery to accelerating clinical trials, enabling highly personalized treatments, and dramatically enhancing healthcare system efficiency, quantum computing holds transformative promise. Its ability to process vast, complex datasets and model biological systems at quantum scales introduces possibilities that were previously unimaginable. As both research and technology continue to evolve, quantum computing will not just support existing methods, it will also transform the core of modern medicine, expanding what is possible in diagnosis, treatment, and healthcare delivery.
